# Severity of motor dysfunction in children with cerebral palsy seen in Enugu, Nigeria

**DOI:** 10.11604/pamj.2017.27.154.11474

**Published:** 2017-06-30

**Authors:** Christian Chukwukere Ogoke, Sylvester Onabeke Iloeje

**Affiliations:** 1 Department of Pediatrics, University of Nigeria Teaching Hospital, Ituku-Ozalla, Enugu, Nigeria; 2 Department of Pediatrics, Enugu State University Teaching Hospital, Parklane, Enugu, Nigeria

**Keywords:** Severity, gross motor dysfunction, GMFCS, Enugu, cerebral palsy, management

## Abstract

**Introduction:**

Children with cerebral palsy (CP) have gross motor dysfunction (GMD) of varying degrees of severity. The Gross Motor Function Classification System (GMFCS) is widely used internationally to classify children with CP into functional severity levels. There are few reports on the use of GMFCS in Nigeria to determine the severity of motor dysfunction in children with CP. This study aims to classify children with CP in Enugu on the basis of severity of their GMD in order to ascertain their management needs.

**Methods:**

The study was a cross sectional observational study and sample selection was by consecutive recruitment. One hundred (100) children with CP aged 9 – 96 months, attending two Pediatric Neurology Clinics in Enugu, were consecutively recruited. Relevant history was taken including modalities of treatment received. Neurological examination was done and the GMFCS manual was used to classify the children into levels of severity.

**Results:**

GMD varied in severity in the patients from mild (47%) (GMFCS levels I & II) to moderate (7%) (GMFCS levels III) and to severe (46%) (GMFCS levels IV & V). Those in levels I – III (54%) were ambulatory while those in levels IV & V (46%) were non-ambulatory. Of the 53 that required mobility assistive device, only 6 (11.3%) were using one.

**Conclusion:**

More than half of CP patients seen in Enugu were ambulatory with mild to moderate motor dysfunction based on the GMFCS. Only a few of our patients are appropriately rehabilitated with augmentative interventions.

## Introduction

Cerebral Palsy (CP) is the most common physical disability in childhood and often places some limitation on a child’s development [1,2]. Cerebral Palsy occurs worldwide with a prevalence of 2-2.5 per 1000 live births in the developed countries and the prevalence has remained stable over the years [1–3]. In developing countries population studies are lacking. In a study of neurology patients in Nigerian hospitals, Izuora and Iloeje [4] in 1989 reported a prevalence of 16% in Enugu while Nottidge and Okogbo [5] in 1991 reported a prevalence of 16.2% in Ibadan. More recent hospital-based studies have reported higher prevalence rates [6–9].

One of the consequences of CP is the limitation of activity such as functional mobility [10]. The classification of motor abilities and limitation of activity (functional classification) is important for a child with CP because it guides management [11, 12]. Children with CP have gross motor dysfunction (GMD) of varying degrees of severity [3]. For ambulatory function, the Gross Motor Function Classification System (GMFCS) is widely used to classify individuals with CP into one of five levels of severity (ambulatory and non-ambulatory status) [1, 11]. The GMFCS is a useful guide to the management needs of patients with CP [1, 11, 13].

There is no standardized classification of the severity of Gross Motor Dysfunction (GMD) in children with CP who attend our PNCs in Enugu. This study was carried out, therefore, to determine severity of motor dysfunction in children with CP using the GMFCS. It is hoped that this will help ascertain their management needs for more independent living.

## Methods

**Study sites:** The study was carried out in the Pediatric Neurology Clinics (PNCs) of University of Nigeria Teaching Hospital (UNTH), Ituku-Ozalla and Enugu State University Teaching Hospital (ESUTH), Parklane. The UNTH, Ituku-Ozalla is one of the first generation Teaching Hospitals in Nigeria, with multidisciplinary departments and caters for patients from predominantly the south-eastern region of the country. The Pediatric Neurology Clinic of UNTH is located in the children out-patient complex. It caters primarily for referred children who have neurological disorders. The Enugu State Teaching Hospital is a smaller and relatively new Teaching Hospital that is also organized into multiple departments.

**Sampling method:** The study was a cross sectional observational (descriptive) study. The sample selection was non-randomized; the subjects were consecutively recruited until the determined sample size was reached. The PNCs of UNTH and ESUTH run weekly on different weekdays.

**Ethical approval and consent:** This research was approved by the University of Nigeria Teaching Hospital Research Ethics Committee and this was accepted by the Enugu State University Teaching Hospital. Informed consent was obtained from the parents/guardians of the patients before recruitment and confidentiality of patient’s information was ensured.

**Study population:** The population studied was CP patients aged 9 to 96 months attending the PNCs of the above teaching hospitals between April 2010 and October 2010. The age bracket (9 months - 96 months) was chosen in view of the age distribution found in a previous study of CP patients in Enugu [14]. In that study, only one patient (0.7%) was older than 8 years [14].

**Inclusion criteria:** All the children aged between 9 and 96 months with a diagnosis of CP were recruited. These cases had history and motor findings consistent with the most recent definition of CP [10,13]. It states that : “Cerebral Palsy (CP) describes a group of permanent disorders of the development of movement and posture causing activity limitation that are attributed to non-progressive disturbances that occurred in the developing fetal or infant brain. The motor disorders of cerebral palsy are often accompanied by disturbances of sensation, perception, cognition, communication and behavior, by epilepsy and by secondary musculoskeletal problems.”

**Exclusion criteria:** Patients with other movement disorders, physical disabilities and motor abnormalities that do not meet the diagnostic criteria for CP, such as muscular dystrophies, paralytic poliomyelitis and spina bifida (myelomeningocele).

**Patient recruitment/enrollment:** Patients who met the inclusion criteria and whose parents/ guardians gave consent were recruited. Each recruited patient was assigned a study identification number and the date of enrolment documented and these were also written boldly on the patient’s folder/case note to prevent multiple enrolment.

**Study procedure:** For each child, socio-demographic data including parental level of education and occupation were ascertained and recorded in the proforma for data collection. Relevant history was obtained including information about the ante-natal, perinatal and postnatal periods and developmental milestones. The gross motor developmental milestones objectively indicated a delay in acquisition of motor milestones in all cases without a regression or loss of already achieved ones. History also indicated in these patients the presence of motor impairment in the first 2-3 years of life and of those impairments known to frequently accompany CP such as visual, speech, cognitive and hearing impairments and epilepsy. The diagnosis of CP was therefore clinical. A consistent history and descriptions of motor findings consistent with CP and the criteria specified in the most recent definition of CP [10, 13] were used to determine case status. A detailed neurological examination adjusted to the age of the child was done. This showed abnormalities of movement, posture, tone and reflexes (deep tendon and primitive) in the recruited patients.

For each child, information on modalities of treatment received (management interventions such as physiotherapy, drugs, use of mobility aids, e.t.c) were obtained and also recorded in the proforma for data collection. The current motor abilities of each child in the home setting were ascertained from the parent /guardian. Subsequently, each child was assessed for his/her motor abilities in the clinic. Each child was assessed for head control and observed in lying, sitting, crawling, standing, walking, running a few meters and climbing stairs. What each child could actually do (present ability) and could not do (limitation) were determined. Using the GMFCS, each child was classified into one of five levels of severity by crosschecking the findings in the appropriate age band of the child.

### Data analysis

Data was analyzed using SPSS version 15. The presentation was in five tables and one figure, showing the distribution of the socio-demographic and other variables. Proportions, means and standard deviations were used to summarize data. The socio-economic class (SEC) was obtained by calculating the mean value (to the nearest whole number) of the scores for the occupation and level of education of the parents or substitutes as recommended by Oyedeji [15]. With this, the subjects were classified into upper class (1 & 11) and lower class (1II-V) [15].

## Results

**Bio-demographic characteristics of study population:** Out of the 100 consecutively recruited patients, there were 58 males and 42 females giving a sex ratio of 1.4:1 (Table 1). The mean age of the children was 32.0 (SD=22.7) months. Mean age of males alone was 27.95 (SD= 18.3) months and of females alone was 37.69 (SD=26.9) months. The distribution of patients by SEC is shown in Table 2.

**Table 1:** Age and sex distribution of patients

Age range (yr)	Gender		Total
	FEMALE N (%)	MALE N(%	
<2yrs	20 (38.5)	32 (61.5)	52
2 - < 4yrs	8 (30.8)	18 (69.2)	26
4 - < 6yrs	6 (60.0)	4 (40.0)	10
6 - 8 yrs	8 (66.7)	4 (33.3)	12
Total	42 (42.0)	58 (58.0)	100

**Table 2:** Distribution of patients by socio economic class

	Frequency N = 100	Relative Freq
SECI	16	0.16
SECII	27	0.27
SECIII	25	0.25
SECIV	30	0.30
SECV	2	0.02

**Figure 1:**
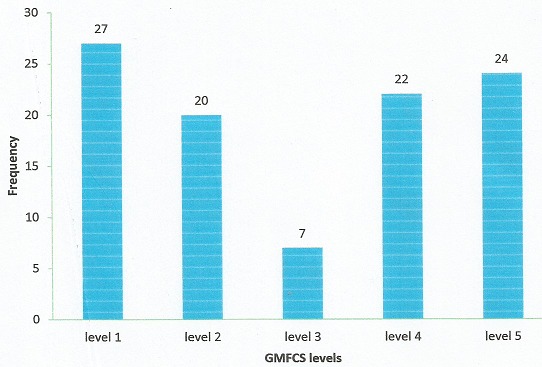
Distribution of GMFCS levels in patients

**Severity of GMD (GMFCS level classification):** The highest proportion of patients (47%) had mild GMD (levels 1 & 2) followed by those with severe GMD (levels 4 & 5) (46%) and the least number (7%) had moderate GMD (level 3) (Figure 1). Fifty-four (54%) patients were ambulatory (GMFCS levels 1, 2 &3) while 46% were non-ambulatory (GMFCS levels 4 & 5) (Table 3, Figure 1).

**Table 3:** GMFCS levels by patient’s age

Age ranged by yr	GMFCS level					Total
	level 1	level 2	level 3	level 4	level 5	
<2yrs	14	10	2	14	12	52
2 - < 4yrs	9	0	3	2	12	26
4 - < 6yrs	2	6	0	2	0	10
6 - 8yrs	2	4	2	4	0	12
Total	27	20	7	22	24	100

**Use of augmentative interventions:** The most commonly employed treatment modality was an impairment-based treatment (Table 4). Only 6 (11.3%) out of the 53 patients with moderate or severe GMD were using mobility assistive devices. Eleven patients (11%) used augmentative intervention and all belonged to the high SEC (Table 5).

**Table 4:** Showing the management interventions for children with CP in Enugu

Intervention	No
Physiotherapy	94
Drugs---oral antispasticity	41
---anticonvulsants	35
Mobility assistive devices	6
Visual aids(spectacles)	5
Orthoses	1
Speech therapy	1
Hearing aids/cochlear implants	Nil
Orthopaedic surgery	Nil
Occupational therapy	Nil
Psychotherapy	Nil
Alternative communication devices	Nil
NG tube/gastrostomy feeding	Nil
Intrathecal Baclofen(ITB)	Nil
Selective Dorsal Rhizotomy(SDR)	Nil

**Table 5:** SEC of children using augmentative interventions

Type of augmentative intervention	No	SEC
Adaptive seating	1	I
Walker	1	I
	2	II
Wheelchair	2	I
Visual aids(spectacles)	4	I
	1	II

## Discussion

The ambulatory function of a child with CP is determined by the severity of GMD. The ambulation and independence of these children are primary concerns for parents and guardians [1]. The GMFCS is widely used internationally to classify children with CP into five functional severity levels that have implications for their management. This study showed that GMFCS level 1 was most common (27%) followed by level V (24%) and level III was least common (7%). Similar observations were made by Howard et al [16] in Victorian study where level 1 was most common (35%) followed by level V (18%) and level III was least common (14%). An important finding from this study was that more than half of the patients (54%) studied were ambulatory belonging to GMFCS levels I – III. This implies that less finance will be expended on mobility aids. This becomes important when it is noted that a greater proportion (57%) of our patients belonged to the lower SEC and cannot afford mobility aids. The reason for this higher number of ambulatory CP patients in our study may be partly because a greater proportion (52%) of the patients studied represents an age group (less than 2 years old) (Table 2) for which using the GMFCS is less precise with a tendency to classifying patients at better functional levels [17].

In comparison to our findings, the study by Vasconcellos et al [18] in Brazil reported lower proportions (34.3%) of ambulatory patients. In addition, Vasconcellos et al [18] studied relatively older patients (4 to 7.5 years) and a smaller sample (n =70). Surprisingly, Nordmark et al [19] in Southern Sweden reported 73% with ambulatory status similar to a study by Howard et al [16] in Australia with a large sample size and large statistical power who reported 65.9% of their 323 children as ambulatory. These studies showed that CP in developed countries is generally milder than in developing countries. Differences in socio-economic status and in the availability of medical care may contribute to these observed variations.

Current management of CP emphasizes the liberal use of mobility assistive devices (MADs) to facilitate independence and societal engagement [1, 11]. One notices that the proportion requiring MADs (53%) is higher than that classified as non- ambulatory (46%). This is because patients classified on level III, though ambulatory, walk with hand-held mobility devices and require wheeled mobility in the community [20]. In practice, the proportion requiring MADs may be higher than reported when we consider that some of the children in this study classified on level II (20%) may need hand-held mobility device when first learning to walk (before 4 years of age) [20]. This suggests that MADs are pivotal in the management of CP.

Only 11.3% of the children studied were appropriately managed with MADs. The reason for this finding may be related to poverty because all patients who were using augmentative interventions belonged to the high SEC (Table 5). Pediatricians should therefore routinely use the GMFCS and counsel parents/ guardians on the need for MADs where applicable. It may be necessary for government to intervene in the provision of augmentative interventions so that poverty will not be a limitationto their use.

## Conclusion

More than half of CP patients aged 9 months to 96 months seen in the neurology clinics in Enugu had mild or moderate motor dysfunction and were ambulatory, based on the GMFCS. Only a few of our patients are appropriately rehabilitated with augmentative interventions. The use of GMFCS helps the clinician to understand the management needs of these children with CP. The GMFCS should be incorporated into routine clinical practice in the management of CP patients.

### What is known about this topic

- CP subtypes vary in severity of gross motor dysfunction;
- Functional classification of CP using the GMFCS assists management;
- Mobility assistive devices are pivotal in management of CP.

### What this study adds

- Gross motor dysfunction in CP may be more severe in resource-poor than in developed countries;
- Patients with CP in resource-poor countries are not commonly rehabilitated with mobility aids.

## Competing interests

The authors declare no competing interests.
